# Successful Management of Cryptococcal Meningitis and Bone Marrow Infiltration in a Young HIV/AIDS Patient: A Case Report and Review of the Literature

**DOI:** 10.1155/2019/1613072

**Published:** 2019-02-19

**Authors:** Sonika Patel, Marla Jalbut, Kimberly S. Esham, David Stone

**Affiliations:** ^1^University of Maryland Medical Center, Internal Medicine, 22 S Greene St., Baltimore, MD 21201, USA; ^2^Tufts Medical Center, 800 Washington St., Boston, MA 02111, USA; ^3^Tufts Medical Center, Hematology/Oncology, 800 Washington St., Boston, MA 02111, USA; ^4^Tufts Medical Center, Infectious Disease, 800 Washington St., Boston, MA 02111, USA

## Abstract

Bone marrow cryptococcosis has been rarely reported in the literature, and there are no established treatment guidelines specific to this AIDS-related complication. The recommended treatment for AIDS-related invasive fungal treatments include amphotericin B and flucytosine which are associated with an array of complications making optimal treatment recommendations difficult. This case presentation represents an example of a patient with newly diagnosed AIDS and bone marrow cryptococcosis, which was successfully managed with an antifungal regimen adjusted to her comorbidities.

## 1. Case Presentation

A 20-year-old woman who recently emigrated from Mexico with limited prior medical care initially presented to an outside hospital with a facial rash thought to be herpes zoster with bacterial suprainfection. She was begun on a course of cephalexin and valacyclovir. Over the following week, she developed new-onset headache, emesis, and photophobia. After returning to the emergency department, she was found to have altered mental status which deteriorated further following a tonic-clonic seizure. A computed tomography scan of the head was unremarkable. Lumbar puncture demonstrated budding yeast and a cryptococcal antigen titer, performed by latex agglutination, that was greater than 1 : 256. During her hospitalization, she was found to be HIV-positive with a CD4 count of 10 cells/*µ*L, a CD4/CD8 ratio of 7%, and an HIV viral load of 37,479 copies/mL.

The cryptococcal infection was initially treated with intravenous liposomal amphotericin 5 mg/kg daily and oral flucytosine 100 mg four times per day upon transfer. After fourteen days, the dose of liposomal amphotericin B was reduced to 4 mg/kg daily due to the onset of acute renal failure (creatinine 1.15 mg/dL from 0.60 mg/dL on admission). Antiretroviral therapy (ART) for HIV was withheld until completion of treatment of cryptococcal meningitis due to risk for immune reconstitution syndrome.

**Figure 1:**
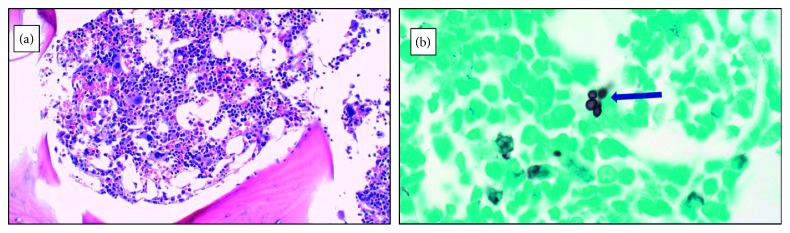
(a) Bone marrow biopsy H&E stain (20x). (b) Bone marrow biopsy GMS stain (100x).

The patient clinically improved on this regimen, with complete resolution of meningeal symptoms within about one week of starting the antifungal regimen. However, soon afterwards, she developed worsening pancytopenia with prominent thrombocytopenia. Platelets were initially 144 k/*µ*L and dropped to a nadir of 46 k/*µ*L. Her white blood cell count decreased to a nadir of 1.8 k/*µ*L (absolute neutrophil count 1100 cells/*µ*L) from 3.4 k/*µ*L and hemoglobin to a nadir of 6.5 g/dL from 9.3 g/dL. During this time, flucytosine was switched to fluconazole due to concern for precipitating worsening thrombocytopenia. Hematology consultation was obtained, and bone marrow aspirate and biopsy showed silver stain positive for disseminated *Cryptococcus neoformans* (Figure 1) with findings of focal phagocytosis, mildly hypocellular bone marrow for age (60% cellularity), and otherwise normal hematopoietic maturation with no evidence of malignancy. Additional negative studies of the bone marrow included acid-fast bacilli (AFB) stain and polymerase chain reaction (PCR) for *Toxoplasma gondii*, Cytomegalovirus (CMV), Parvovirus B19, and *Bartonella*. *Histoplasma* was not directly noted on histopathologic analysis of the bone marrow. The patient tested negative for CMV in the bone marrow but was positive for <1000 copies/mL of CMV in the peripheral blood. No treatment for CMV was administered.

Liposomal amphotericin B and fluconazole 800 mg daily were continued for a total of four weeks of induction therapy in the setting of *Cryptococcus* in the bone marrow and associated pancytopenia. Pancytopenia improved with continuation of this regimen. At the time of discharge to a long-term care facility, her white blood cell count was 2.4 k/*µ*L, hemoglobin was 8.6 g/dL, and platelets had improved to 80 k/*µ*L. The patient was started on abacavir/dolutegravir/lamivudine after two negative lumbar punctures. Given persistently elevated serum *Cryptococcus* Ag > 1 : 256, the patient was continued on suppressive fluconazole (200 mg daily) therapy for the next two years.

## 2. Methods and Results

We searched PubMed using the terms “bone marrow cryptococcosis AND HIV” and performed a citation review of each paper. We identified seven English language case reports published since 1990 that specifically reported patients positive for *Cryptococcus* in the bone marrow [1–7]. Two studies were excluded as they were case reports of non-HIV patients, and two were excluded as they did not specify the cryptococcosis treatment regimen [1, 2, 5, 6]. We summarize the key features of three cases of *Cryptococcus* infection of the bone marrow in the literature in addition to this case report.

## 3. Discussion & Conclusions

Three single-patient case reports addressed the treatment used for bone marrow cryptococcosis among HIV patients (Table 1) [1–3, 8, 9]. All three of these patients were treated according to variations in the guidelines for cryptococcal meningitis which include a two-week induction phase with amphotericin B and flucytosine or amphotericin B and fluconazole, depending on the availability and tolerance of flucytosine, and an eight-week antifungal consolidation phase [7]. Two of these case reports had patients who survived to discharge [2, 3].

This case report represents the third reported case of a patient with concomitant HIV and bone marrow cryptococcosis to survive past hospitalization with treatment.

The remaining case summarized in Table 1 involved a 46-year-old man with HIV and concomitant histoplasmosis infection of the bone marrow who did not survive [2]. This patient's older age at diagnosis, possible advanced stage of his multiple comorbidities, and concomitant histoplasmosis infection may have led to his death despite treatment, whereas the two reported cases of survivors past hospitalization and the present case were all less than 21 years old at diagnosis.

This is the first case report to address medication dosing and duration for a patient with concomitant HIV and disseminated cryptococcosis in the setting of pancytopenia, with prominent thrombocytopenia (platelets 46 k/*µ*L). It is likely the patient's thrombocytopenia was secondary to a combination of both the antifungal therapy and bone marrow cryptococcosis, as evidenced by the recovery of hematopoiesis with adjustment of antifungal therapy from flucytosine to fluconazole. The successful management of this patient demonstrates the importance of tailoring antifungal therapy to a patient's comorbidities. The patient was successfully treated on less than maximal dosage of liposomal amphotericin B for a longer duration of 4 total weeks and second-line fluconazole, in lieu of first-line flucytosine. Future studies should report the specific antifungal therapy dosages and duration in patients of various ages and comorbidities with bone marrow cryptococcosis to better identify optimal treatment regimens.

This case presentation should alert clinicians to consider cryptococcal involvement of the bone marrow in the differential diagnosis of severely immunocompromised patients with HIV and pancytopenia. Although this condition is rare, early diagnosis is crucial to the survival of these patients. This case should also highlight the need to individualize treatment regimens for bone marrow cryptococcosis based on patients' comorbidities and to report these specific medication regimens in the literature.

## Figures and Tables

**Table 1:** Reported treatments for *C. neoformans* infection in the bone marrow of HIV patients.

Study	Patient demographics	Diagnosis	Treatment for cryptococcosis	Survival to hospital discharge?
Serrano Ocana et al [4]	20-year-old African American female	HIV, *C. neoformans* meningitis, and *C. neoformans* in bone marrow	Amphotericin B^*∗*^ 0.8 mg/kg/day IV for 2 weeks and fluconazole 10 mg/kg/day for 8 weeks	Yes
Friedman et al [5]	12-year-old Thai male	HIV, *C. neoformans*, and *Salmonella* enteritis group D in bone marrow	Amphotericin B 1 mg/kg/day for 2 weeks and fluconazole 12 mg/kg/day for 8 weeks	Yes
Swaminathan et al [3]	46-year-old Hispanic male	HIV, *Histoplasma*, and *C. neoformans* in bone marrow	Amphotericin B 1 mg/kg/day IV	No

*C. neoformans*: *Cryptococcus neoformans*. ^*∗*^Amphotericin B deoxycholate formulation was used in all studies above.

## Abbreviations

HIV/AIDS: Human immunodeficiency virus/acquired immunodeficiency syndrome
ART: Antiretroviral therapy
AFB: Acid-fast bacilli
PCR: Polymerase chain reaction
CMV: Cytomegalovirus
*C. neoformans*: *Cryptococcus neoformans*.
